# Transgenic sugarcane harboring *cry1Ab* and *vip3Aa* Bt genes for the management of *Diatraea saccharalis*

**DOI:** 10.3389/fpls.2026.1887829

**Published:** 2026-08-11

**Authors:** Florencia Budeguer, Ramón Enrique, Florencia García Degano, Santiago Ostengo, Aldo Sergio Noguera, María Francisca Perera, Josefina Racedo

**Affiliations:** 1 Instituto de Tecnología Agroindustrial del Noroeste Argentino, Estación Experimental Agroindustrial Obispo Colombres - Consejo Nacional de Investigaciones Científicas y Técnicas, Las Talitas, Tucumán, Argentina; 2 Estación Experimental Agroindustrial Obispo Colombres (EEAOC), Las Talitas, Tucumán, Argentina

**Keywords:** *Bacillus thuringiensis*, Cry proteins, sugarcane borer, gene pyramiding, genetic transformation, insect resistance, Vip proteins

## Abstract

The sugarcane borer (*Diatraea saccharalis*) is a major pest of sugarcane in the Americas, causing significant yield and quality losses. Genetic transformation using *Bacillus thuringiensis* (Bt) genes represents an effective strategy for integrated pest management. In this study, *cry1Ab* and *vip3Aa* genes were co-introduced into embryogenic calli of the commercial sugarcane variety TUC 03–12 using microprojectile bombardment to generate pyramided Bt events. Transgene integration was confirmed by PCR, with 247 regenerated lines testing positive for at least one transgene. Based on the total number of bombarded embryogenic calli, the overall transformation efficiency was 6.24%. Protein expression screening using lateral flow immunoassays showed that 76.6% of PCR-positive lines expressed detectable Cry1Ab protein, whereas Vip3Aa protein was detected in 33.3% of the double-positive lines. Phenotypic bioassays under controlled conditions demonstrated high larval mortality of *D. saccharalis*, exceeding 80% in several transgenic lines expressing both proteins. Quantification of Cry1Ab protein by ELISA revealed variability among independent lines and supported the functional relevance of protein accumulation for insect resistance. RT-qPCR analysis showed 3–140-fold and 2–1400-fold increases in *cry1Ab* and *vip3Aa* transcript levels, respectively, compared with non-transformed plants. A strong positive correlation was observed between *cry1Ab* transcript levels and protein accumulation, indicating that transcriptional activity is a major determinant of toxin levels. These results demonstrate that several transgenic lines exhibit enhanced resistance to *D. saccharalis* under controlled conditions, including lines expressing both Cry1Ab and Vip3Aa proteins. These findings support the potential of pyramided Bt sugarcane as a promising strategy for *D. saccharalis* management.

## 1 Introduction

The sugarcane borer (*Diatraea saccharalis*; Lepidoptera: Crambidae) is one of the most destructive pests of sugarcane in the Americas, including Argentina, where it causes significant yield and quality losses (Ostengo et al., 2022). Early instar larvae feed on leaf tissues, while later instars bore into the stalks, disrupting plant vascular integrity. Larval tunneling also facilitates the entry of fungi and bacteria associated with the red rot disease complex, further reducing sugar and ethanol production (Perez et al., 2024). Conventional management strategies for *D. saccharalis*, including cultural practices, biological control, and chemical insecticides, often provide incomplete control and may entail environmental and economic costs.

Transgenic crops expressing insecticidal proteins from *Bacillus thuringiensis* (Bt) have become an effective and environmentally compatible tool for pest management due to their high specificity and safety for non-target organisms. Bt produces Cry proteins during sporulation and Vip proteins during the vegetative phase; both act on the larval midgut but bind to distinct receptors. The lack of structural homology and the use of independent binding sites reduce the likelihood of cross-resistance between Cry and Vip proteins and support their combined use in transgenic crops (Baranek et al., 2017; Carrière et al., 2016; Chakroun et al., 2016; Qamar et al., 2021; Wang et al., 2025).

While Cry proteins have been extensively studied and widely deployed in transgenic crops, Vip proteins remain comparatively underexplored, particularly in complex polyploid crops such as sugarcane. Most studies on Vip3A have been conducted in model or alternative crops such as tobacco and cotton (Kalia and Kaur, 2019; Khan et al., 2020), leaving a gap in our understanding of its expression stability, protein accumulation, and contribution to insect resistance in sugarcane. In addition, although Vip proteins are active against a broad range of lepidopteran pests, their effectiveness against *D. saccharalis* has been less extensively documented than that of Cry proteins, highlighting the need for further evaluation in this target species.

Modern sugarcane cultivars are highly polyploid and aneuploid interspecific hybrids derived primarily from *Saccharum officinarum* and *Saccharum spontaneum*, possessing large and complex genomes with variable chromosome numbers and extensive genomic redundancy (D'Hont et al., 1996; Garsmeur et al., 2018). This genetic complexity represents an additional challenge for transgene expression that may vary considerably among independent events due to position effects, copy number variation, epigenetic modifications, and gene silencing mechanisms (Kohli et al., 2003). Post-transcriptional gene silencing has been reported in transgenic sugarcane, affecting transgene accumulation and expression stability (Ingelbrecht et al., 1999; Birch et al., 2010).

Although Bt crops have been widely adopted across diverse agricultural systems, field-evolved resistance to several Cry proteins has been documented in important target pests, including *Spodoptera frugiperda* in Brazil and *Helicoverpa zea* in the United States (Tabashnik et al., 2013; Trumper, 2014; Bernardi et al., 2015; Dively et al., 2016). To delay the development of resistance, strategies such as high-dose expression, refuge implementation, and the deployment of crops expressing multiple Bt toxins (gene pyramiding) have been proposed and adopted, including in sugarcane (Cristofoletti et al., 2018; Wang et al., 2025). In major crops such as maize and cotton, pyramided Bt events have demonstrated improved durability and efficacy. In sugarcane; however, the commercial deployment of Bt technology remains limited. Since 2017, Bt sugarcane cultivars expressing single Cry proteins have been commercially cultivated in Brazil for the control of *D. saccharalis* (de Oliveira et al., 2022; CTC, 2025). Currently, only a few Bt sugarcane cultivars have been approved for cultivation, including CTC20BT, CTC9001BT, CTC7515BT, and CTC9003BT, all of which rely on a single Cry protein (CTC, 2025). To date, no sugarcane variety expressing pyramided Bt genes has been commercially released. The development of sugarcane lines combining *cry1Ab* and *vip3Aa* genes, therefore, represents a promising strategy to improve pest management and to advance the next generation of insect-resistant cultivars.

In this study, we generated transgenic events of the Argentine commercial sugarcane variety TUC 03–12 expressing pyramided *cry1Ab* and *vip3Aa* genes via biolistic transformation. TUC 03–12 is a commercially important cultivar developed by the Estación Experimental Agroindustrial Obispo Colombres (EEAOC), characterized by high productivity and favorable sanitary performance (Cuenya et al., 2015). This has favored its rapid adoption by sugarcane growers in northwestern Argentina, where it is currently the second most widely cultivated variety, accounting for 18.6% of the planted area (Henríquez et al., 2025). Transgene integration and expression were evaluated, and resistance of selected events to *D. saccharalis* was assessed under controlled conditions.

## 2 Materials and methods

### 2.1 Callus induction

Embryogenic calli were induced from the sugarcane variety TUC 03-12, developed by the Estación Experimental Agroindustrial Obispo Colombres (EEAOC, Tucumán, Argentina). Immature leaf roll explants were excised from the upper internodal region of sugarcane stems and used as source material for embryogenic callus induction. Transverse sections (~1 mm thick) were obtained from the central whorl region above the apical meristem and cultured on callus induction medium as described by Bower and Birch (1992). Embryogenic callus induction was performed using A+3 medium containing Murashige and Skoog (MS) basal salts (4.33 g L^−1^) supplemented with 10 mL L^−1^ of a vitamin solution for callus culture, 3 mg L^−1^ 2,4-dichlorophenoxyacetic acid (2,4-D), 0.1 g L^−1^ myo-inositol, 0.5 g L^−1^ casein hydrolysate, 0.2 g L^−1^ citric acid, 0.05 g L^−1^ arginine, and 20 g L^−1^ sucrose. The medium was solidified with 6 g L^−1^ agar (Sigma-Aldrich). The pH was adjusted to 5.7–5.8 before autoclaving. After sterilization and cooling to approximately 50°C, gentamicin (16 mg L^−1^) and cefotaxime (100 mg L^−1^) were added to reduce microbial contamination. Cultures were incubated in the dark at 26 ± 2°C for four weeks to promote embryogenic callus formation.

### 2.2 Genetic constructs and gene transfer

Transformations were performed using three plasmids: one carrying the *cry1Ab* gene (under the control of the Zm-UBI1 promoter), another carrying the *vip3Aa* gene (under the control of the Zm-UBI1 promoter), and a third carrying the *nptII* gene (under the control of the Actin promoter). The *cry1Ab* and *vip3Aa* genes encode insecticidal proteins derived from *Bacillus thuringiensis* (Bt). The *nptII* gene encodes neomycin phosphotransferase II, which confers resistance to geneticin and is used as a selectable marker. For particle bombardment, the three purified plasmids were co-precipitated onto microcarriers following the protocol described by Altpeter and Sandhu (2010). Bombardment was performed using a Biolistic^®^ PDS-1000/He system (Bio-Rad Laboratories, USA), according to the manufacturer’s instructions.

### 2.3 Selection, regeneration, and micropropagation conditions

Transformed calli were selected on culture medium containing 50 mg L^-1^ geneticin. Selected shoots were regenerated on medium supplemented with 0.7 mg L-1 6-benzylaminopurine (BAP) under a 16-h photoperiod (Noguera et al., 2015). Regenerated shoots were subsequently micro propagated and rooted following a previously optimized protocol (Noguera et al., 2010) and acclimated under controlled conditions (2500 lux and 80 - 100% humidity) before transferred to *ex vitro* conditions.

### 2.4 Detection of transgenic events by PCR

Total genomic DNA was extracted from 100 mg of young leaf tissue from putative transgenic sugarcane lines resistant to geneticin, as well as from the commercial Bt maize hybrid SYN 126 VIPTERA3 (NK Semillas, 2025) (positive control) and non-transgenic TUC 03–12 plants (negative control). DNA isolation was performed using the CTAB method described by Aljanabi et al. (1999). DNA concentration was determined by UV spectrophotometry, and purity was assessed using the A260/A280 ratio. DNA integrity was verified by electrophoresis on 1% (w/v) agarose gels. PCR amplification of *cry1Ab* and *vip3Aa* transgenes was performed using gene-specific primers (**Table 1**). Reactions were carried out in a total volume of 20 µL containing 50 ng of genomic DNA, 2 µL 10× PCR buffer (without MgCl_2_), 2 µL MgCl_2_ (25 mM), 0.8 µL dNTP mix (10 mM each), 0.5 µL of each primer (20 µM), 0.2 µL Taq DNA polymerase (Invitrogen), and nuclease-free water to a final volume. Thermal cycling conditions were as follows: initial denaturation at 95°C for 3 min; 30 cycles of 95°C for 1 min, 59°C for 45 s, and 72°C for 1 min; followed by a final extension at 72°C for 5 min. PCR products were separated on 1% (w/v) agarose gels in 1× TAE buffer, stained with GelRed (Biotium), and visualized under UV light. Expected amplicon sizes were 500 bp for *cry1Ab* and 493 bp for *vip3Aa*. PCR analyses were performed at least twice for each gene to confirm reproducibility. Transformation efficiency was calculated as the percentage of transgenic lines positive for at least one gene of interest relative to the total number of bombarded calli.

**Table 1:** Primer sequences and expected amplicon sizes used for PCR analyses.

Gene	Primer	Sequence (5′–3′)	Amplicon size (bp)
*cry*	Forward	CAGTTCAGGAGAGAATTGACC	500
	Reverse	GGCAAGTTAGAAGAGGTTCC	
*vip*	Forward	CGTGTACAACTTCCTGATCG	493
	Reverse	CAGGGTCTTCATCTTCTTGG	

### 2.5 Detection of Cry1Ab and Vip3Aa proteins by lateral flow immunoassay

Detection of Cry1Ab and Vip3Aa proteins in transgenic sugarcane lines was performed using AgraStrip^®^ lateral flow immunoassay kits specific for Cry1Ab and Vip3Aa Bt proteins (Romer Labs, Austria), following the manufacturer’s instructions. Young leaf tissue from *in vitro*-grown plants was collected from each PCR-positive line. For each transformation event, a pooled sample (50 mg) from all regenerated plants was used to obtain a representative extract. Non-transformed TUC 03–12 plants served as the negative control, whereas the commercial Bt maize hybrid SYN 126 VIPTERA3 was used as the positive control. Leaf tissue was homogenized in 500 µL of extraction buffer provided with the kit (1:10, w/v) and incubated for 5 min at room temperature. The test strip was immersed in the homogenate and incubated for 5 min at room temperature, after which the results were recorded. The presence of the test line (T) and a control line (C) was interpreted as a positive result. In contrast, the presence of only the control line indicated a negative result. Tests lacking the control line were considered invalid and repeated. Each extract was analyzed in duplicate to ensure reproducibility, and positive and negative controls were included in each assay batch to verify test performance. The proportion of lines expressing detectable Cry1Ab or Vip3Aa proteins was calculated relative to the total number of PCR-positive lines for each gene.

### 2.6 Phenotypic assays against *D. saccharalis* under controlled conditions

*Diatraea saccharalis* larvae were obtained from a laboratory colony maintained at the Entomology Department of the Estación Experimental Agroindustrial Obispo Colombres (EEAOC). The colony was originally established from sugarcane field populations collected in January 2025 in Overo Pozo County (S: 26° 50′ 4.4′′, W: 64° 52′ 8′′), Tucumán, Argentina, and subsequently maintained for several generations under controlled laboratory conditions. Larvae were reared in environmental chambers at 26 ± 2°C, 70 ± 10% relative humidity, and a 14:10 h light:dark (L:D) photoperiod on an artificial diet, following the protocol of Fogliata et al. (2016). All emerged adults were examined using taxonomic morphological markers (Dyar and Heinrich, 1927) to confirm the species identity. Bioassays were conducted using 128-well Pitman trays (Ghimire et al., 2018). Each well was filled with 4% (w/v) sterile agar, supplemented with 0.16 g L^−1^ ascorbic acid and 0.16 g L^−1^ streptomycin prior to solidification to reduce tissue oxidation and microbial contamination. After solidification, a leaf fragment from each transgenic line was placed on the agar surface, and one first-instar (L1) larva of *D. saccharalis* was introduced per well. Larval mortality was recorded after four days, and corrected using Abbott’s formula (Abbott, 1925) to account for natural mortality in the negative control. All assays were conducted under controlled environmental conditions of 26 ± 2°C, 70 ± 10% relative humidity, and a 14:10 h light:dark photoperiod.

Three types of controls were included in each assay: (i) a regenerated event lacking detectable transgene integration (PCR-negative) (L22), (ii) the wild-type genotype TUC 03-12, both used as negative controls, and (iii) the Bt maize hybrid SYN 126 VIPTERA3 expressing Cry/Vip proteins, used as positive controls. In the initial screening assay, 134 transgenic lines were evaluated, each with three biological replicates, to identify events with increased resistance to *D. saccharalis*. Based on the mortality results from the first screening, 60 lines were classified into the highest-resistance group (Group A). From this subset, 18 lines with different transgene combinations, including single (*cry1Ab* or *vip3Aa*) and pyramided (*cry1Ab* + *vip3Aa*) events, and exhibiting phenotypic characteristics similar to the parental variety TUC 03–12 were selected for further evaluation. For each transgenic line, leaf samples collected from all available plants were pooled before the bioassay. Each replicate consisted of 16 neonate larvae individually exposed to leaf fragments from the pooled sample in a separate tray. Three replicates were evaluated per line in the initial screening assay and six replicates per line in the second assay to improve the robustness and statistical power of the analysis. Mortality data were analyzed using a linear model (LM) with Bt line included as a fixed in NAVURE software (Navure Team, 2023). Differences among means were assessed using the DGC (Di Rienzo, Guzmán, and Casanoves) *post-hoc* test (p < 0.05). Graphical representations were generated in R (RStudio, version 2026.01.2) using the ggplot2 package.

### 2.7 Protein quantification by ELISA

Cry1Ab protein accumulation in plant tissue was quantified using an enzyme-linked immunosorbent assay (ELISA). Fresh leaf samples from transgenic sugarcane lines and control plants were processed to obtain protein extracts following standard procedures. Absorbance was measured using a microplate reader, and optical density (OD) values within the linear range of the assay (0.1–1.4) were retained for analysis. Cry1Ab protein concentration was estimated using a calibration curve provided with the ELISA assay. Values were corrected according to the corresponding dilution factor and initially expressed in parts per billion (ppb), then converted to nanograms per gram of fresh tissue (ng g^−1^). Measurements were performed in triplicate (n=3), and results are presented as mean ± standard error for each sample. Data processing and graphical visualization were performed in R (RStudio, version 2026.01.2) using the readxl, dplyr, and ggplot2 packages.

### 2.8 Quantitative reverse transcription PCR (RT-qPCR) analysis

RT-qPCR was performed on a subset of transgenic lines previously characterized by PCR and protein assays to estimate *vip3Aa* transcript levels as an indirect measure of protein accumulation, due to the lack of a purified Vip3Aa standard for ELISA quantification. Total RNA was extracted from young leaves of six selected transgenic sugarcane lines harboring both *cry1Ab* and *vip3Aa* transgenes, together with non-transformed TUC 03–12 plants, using the SV Total RNA Isolation Kit (Promega, USA) according to the manufacturer’s instructions. RNA quality was assessed by electrophoresis on 1% (w/v) agarose gels and by A260/A280 absorbance ratio. Complementary DNA (cDNA) was synthesized from 1 µg of DNase-treated total RNA using M-MLV Reverse Transcriptase (Promega, USA) and oligo(dT)_15_ primers, following the manufacturer’s protocol. Gene-specific primers for *cry1Ab*, *vip3Aa*, and the reference *actin* gene were described by Budeguer et al. (2024). Relative transcript levels were calculated using the 2^−ΔΔCt^ method (Livak and Schmittgen, 2001), where ΔΔCt = ΔCt (sample) – ΔCt (calibrator). Expression values were normalized to *actin* as the endogenous control. RNA of non-transformed TUC 03–12 plants was used as the calibrator (reference sample) for the 2^−ΔΔCt^ analysis and were assigned a relative expression value of 1. Data were analyzed using a linear model implemented in NAVURE v1.2.0 software (Navure Team, 2023), an identity variance function (varIdent) was incorporated into the model. Mean comparisons were performed using the DGC *post-hoc* test (p < 0.05). Correlation analysis between *cry1Ab* transcript levels and protein accumulation was assessed using Pearson’s correlation coefficient. Graphs were generated in R using the *ggplot2* package.

## 3 Results

### 3.1 Transformation, selection, and molecular detection of transgenic events

**Figure 1:**
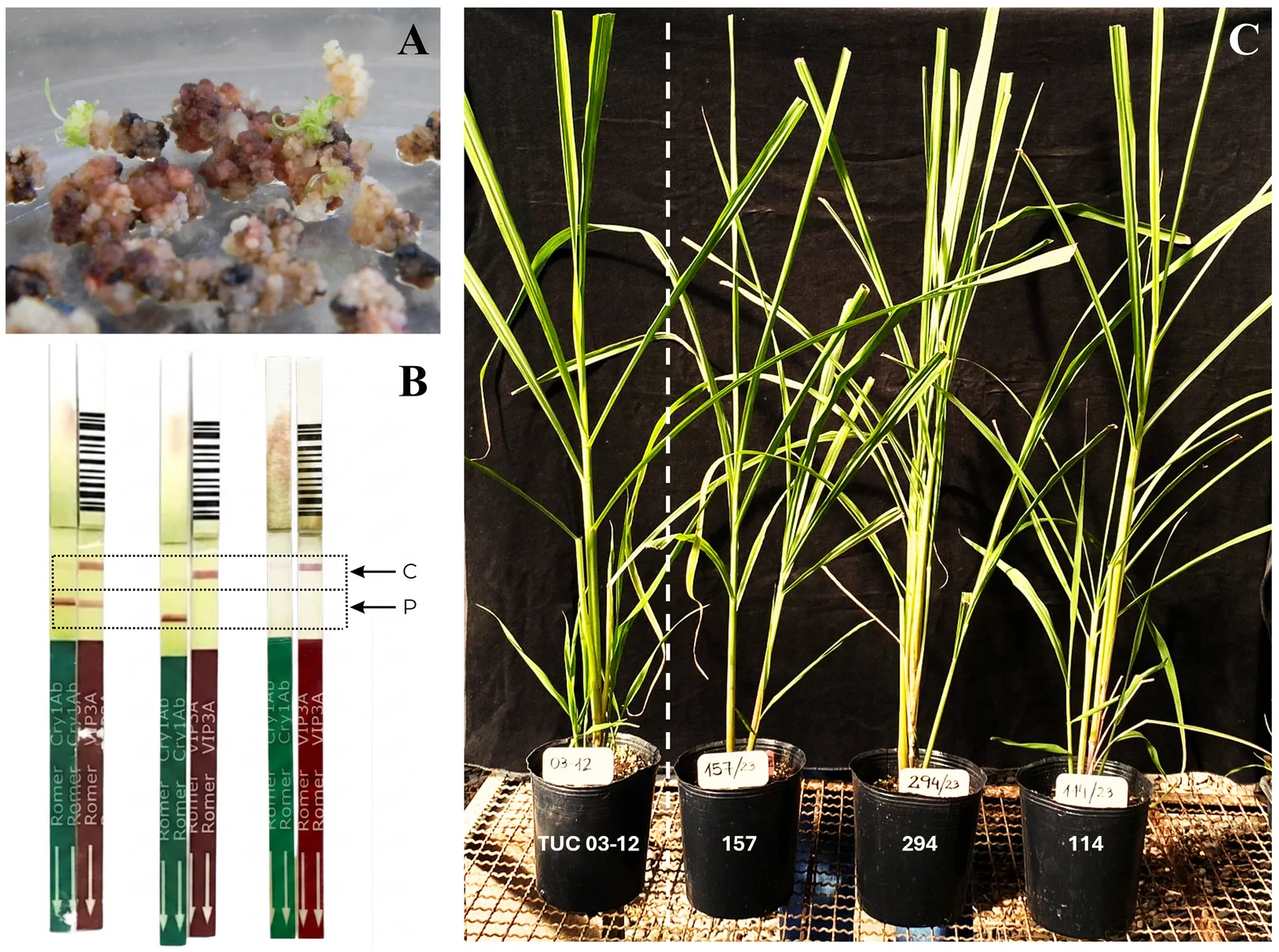
Regeneration, molecular screening, and characterization of transgenic sugarcane plants expressing Cry and Vip proteins. **(A)** Sugarcane calli after genetic transformation and selection in medium containing geneticin, showing regeneration of antibiotic-resistant plants. **(B)** Detection of Cry and Vip proteins in regenerated plants by lateral flow immunoassays. The control band C is present in all samples, while the positive band P indicates Cry (green device) or Vip (red device) detection, allowing the identification of pyramided Cry+Vip, single-Cry and non-transformed lines. **(C)** Phenotypic characterization of wild-type control plants (TUC 03-12) and transgenic lines expressing pyramided Cry and Vip proteins.

A total of 374 geneticin-resistant lines were recovered from 3,960 bombarded embryogenic calli (**Figure 1A**) and subsequently screened by PCR for the presence of the *cry1Ab* and *vip3Aa* transgenes. PCR analysis identified 247 positive lines; 229 lines carrying only the *cry1Ab* gene and 18 carrying both *cry1Ab* and *vip3Aa* genes, resulting in an overall transformation efficiency of 6.24%. No lines carrying *vip3Aa* alone were detected. Among the PCR-positive events, 171 lines (162 *cry1Ab*-positive and 9 *cry1Ab* + *vip3Aa*) were successfully rooted and acclimated under greenhouse conditions and subsequently used for protein detection and phenotypic bioassays. The nine pyramided (*cry1Ab* and *vip3Aa*) lines corresponded to events L30, L113, L114, L157, L177, L294, L357, L440, and L573.

### 3.2 Detection of Cry1Ab and Vip3Aa proteins by lateral flow assay

The presence of Bt proteins was assessed in the 171 PCR-positive lines using lateral flow immunoassays. Representative results of Cry1Ab and Vip3Aa protein detection are presented in **Figure 1B**. Cry1Ab protein was detected in 131 lines, representing 76.60% of the evaluated events. Among the pyramided (*cry1Ab* + *vip3Aa*) lines, three (L114, L157, and L294) showed detectable levels of both Cry1Ab and Vip3Aa protein (33.3%) (**Figure 1C**).

### 3.3 Phenotypic assays against *D. saccharalis* under controlled conditions

Larval mortality of *D. saccharalis* feeding on transgenic sugarcane lines was evaluated under controlled conditions and corrected using Abbott’s formula. As expected, the conventional TUC 03–12 plants showed mortality below 20% across all assays. In contrast, the Bt maize control exhibited a corrected efficacy of 96.78%, confirming the reliability of the bioassay conditions.

**Figure 2:**
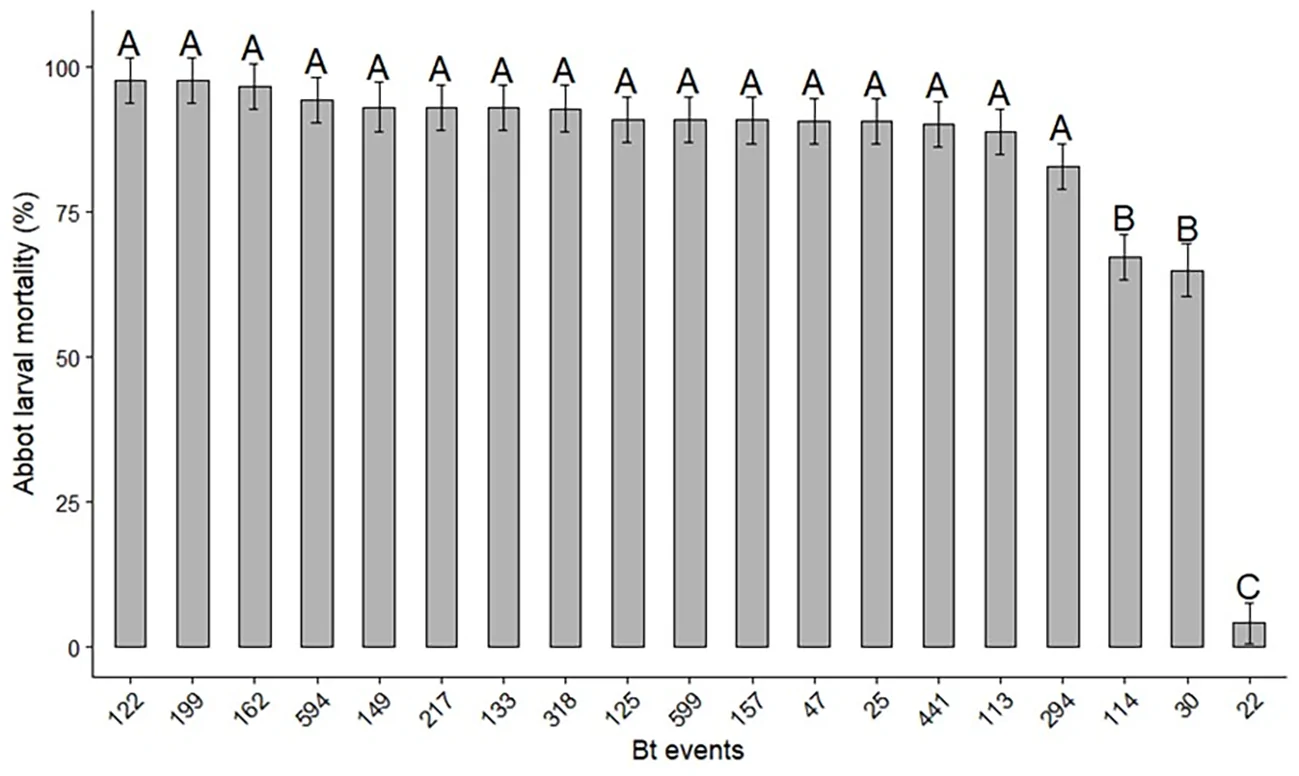
Corrected larval mortality (%) of transgenic sugarcane lines in response to *Diatraea saccharalis* under controlled conditions. Mortality was calculated using Abbott’s formula. Eighteen transgenic lines and one non-transgenic regenerated control (line L22) were evaluated. Different letters indicate significant differences among treatments according to the DGC test (p < 0.05). Error bars represent standard errors derived from the linear model (LM).

**Figure 3:**
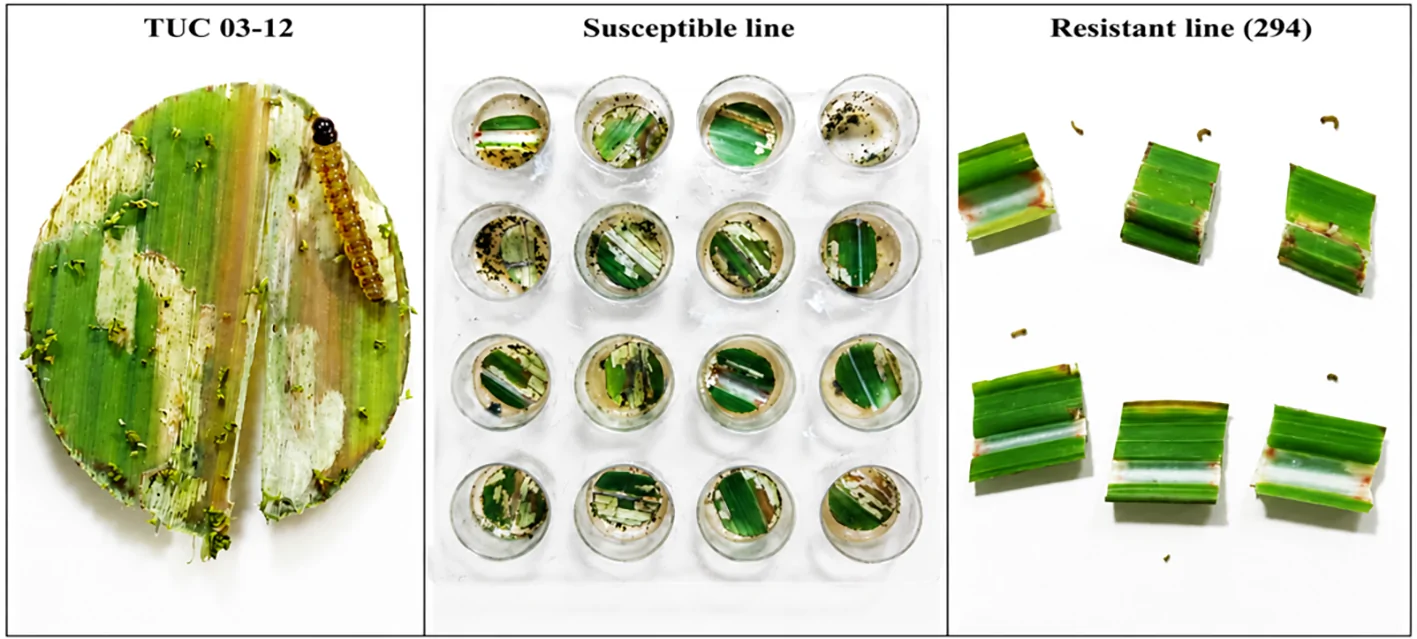
Representative feeding damage caused by *Diatraea saccharalis* larvae on sugarcane leaves after four days under controlled conditions. Leaves from control plants (TUC 03-12) and susceptible lines showing extensive feeding damage and the presence of live larvae. Leaves from a resistant transgenic line (L294) showing minimal tissue damage and dead larvae.

In the initial screening assay, 134 transgenic lines were evaluated (**Supplementary Figure 1**). Based on corrected mortality values, 60 lines were classified within the highest resistance group (Group A). From this subset, 18 selected lines with different transgene combinations and phenotypic characteristics similar to the parental variety TUC 03–12 were further evaluated in a second assay with increased biological replication. Among these lines, 16 showed high larval mortality rates ranging from 83% to 98% (Group A), including two pyramided events with both Bt proteins detected (L157 and L294) (**Figure 2**). Lines L114 and L30 exhibited intermediate efficacy (Group B), with mortality values of approximately 65–70%. The non-transgenic regenerated control (L22) showed the lowest efficacy, with a mortality of 2.89%, and differed significantly from all other treatments, being assigned to Group C. Representative feeding damage caused by *D. saccharalis* larvae on leaves from the non-transgenic regenerated control (L22) and selected transgenic lines after four days of exposure is shown in **Figure 3**.

### 3.4 Cry1Ab protein accumulation in sugarcane lines

**Figure 4:**
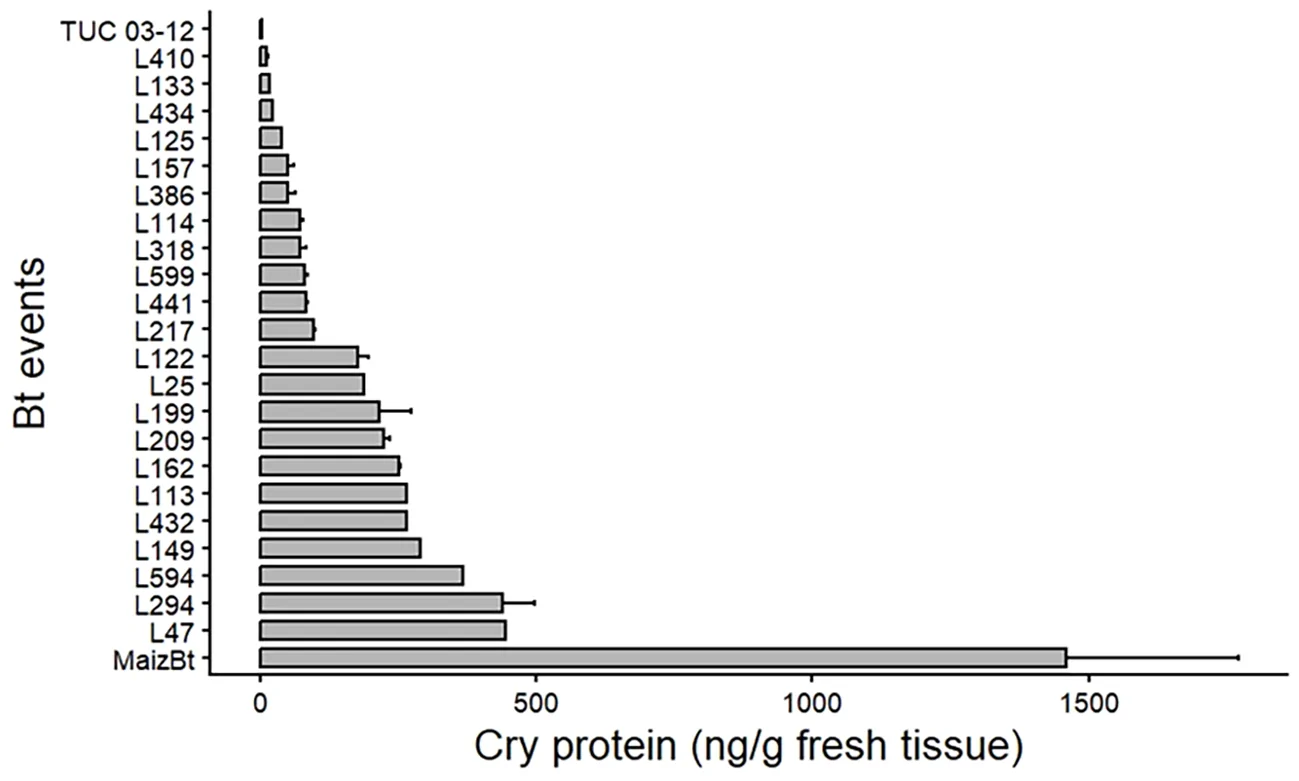
Cry protein content (ng g^−1^ fresh weight) in transgenic sugarcane lines determined by ELISA. Bt maize and non-transgenic sugarcane (TUC 03-12) were included as positive and negative controls, respectively. Values are presented as mean ± SE.

Cry1Ab protein accumulation varied markedly among transgenic sugarcane lines (**Figure 4**). The Bt maize control showed the highest protein levels, exceeding 1400 ng g^−1^ fresh weight (FW), confirming the sensitivity and reliability of the assay. Among the transgenic events, line L47 and L294 exhibited the highest Cry1Ab protein accumulation (approximately 450–500 ng g^−1^ FW), whereas lines L434, L133, and L410 showed the lowest levels, with values below 100 ng g^−1^ FW. As expected, the non-transgenic control (TUC 03-12) showed negligible Cry1Ab protein levels. Overall, these results reveal substantial variability in Cry1Ab protein accumulation among independent transformation events, suggesting differences in transgene expression across lines.

### 3.5 Transgene expression assessed by RT-qPCR

**Figure 5:**
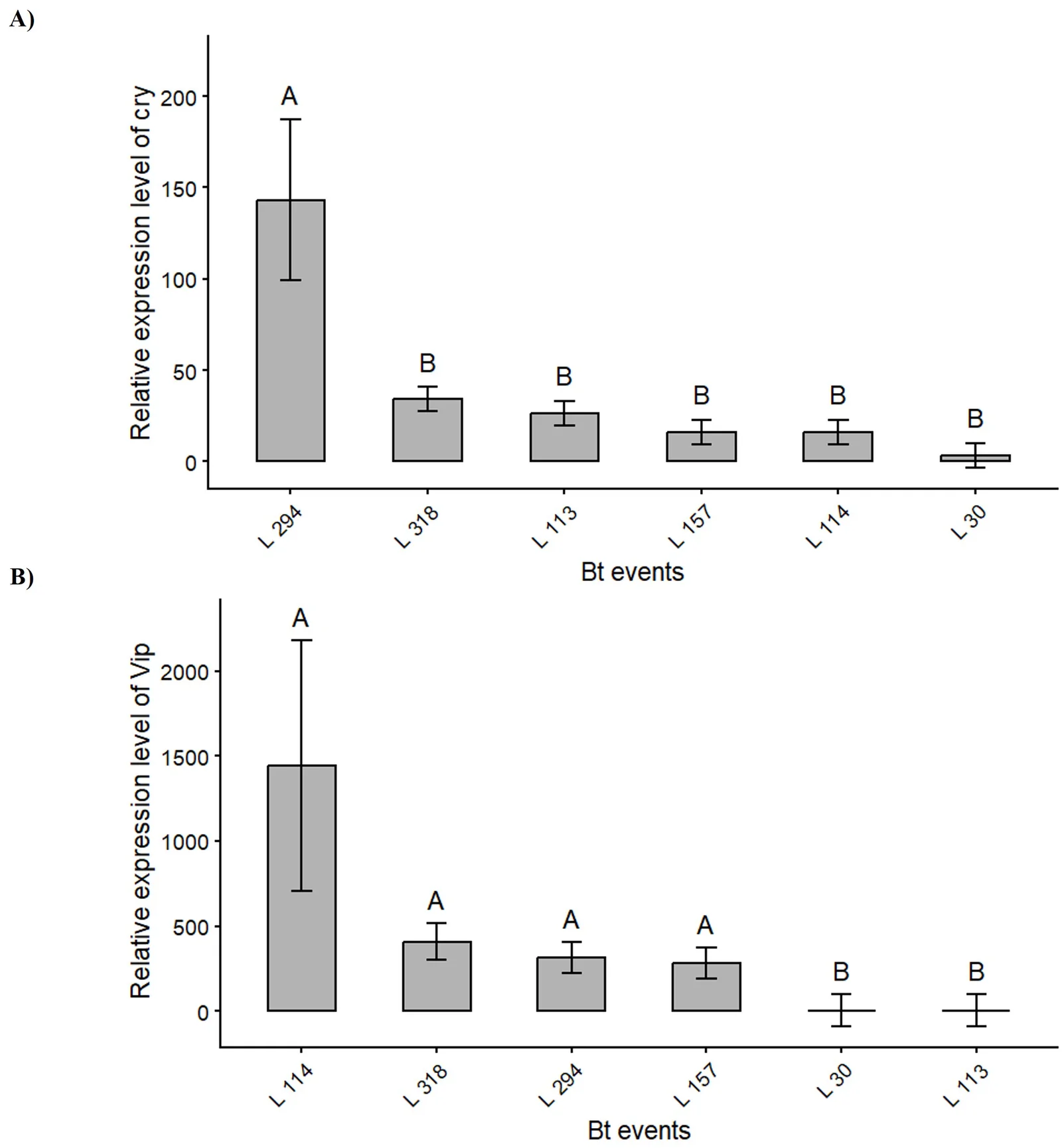
Relative expression of *cry* and *vip* transcripts in transgenic sugarcane lines determined by RT-qPCR. **(A)** Relative expression of the *cry* gene in six independently transformed lines compared with non-transformed TUC 03–12 plants. **(B)** Relative expression of the *vip* gene in six pyramided (*cry* and *vip*) lines. Relative expression values were calculated as mean 2^−ΔΔCt^ ± SE. The non-transformed TUC 03–12 plants were used as the calibrator (expression level = 1). Different letters indicate statistically significant differences according to the DGC test (p < 0.05).

RT-qPCR analysis was conducted on a subset of lines, including six pyramided events (*cry1Ab* and *vip3Aa*) previously identified by PCR. Line 294 exhibited the highest *cry1Ab* transcript accumulation (143.09-fold) and showed high *vip3Aa* expression (313.63-fold) relative to the non-transformed control TUC 03–12 plants (**Figure 5**). Across the remaining lines, *cry1Ab* expression ranged from 3- to 34-fold (**Figure 5A**). For the *vip3Aa* gene, relative expression levels varied widely, ranging from 2- to 1,443.9-fold (**Figure 5B**). Four lines exhibited markedly higher expression levels (Group A), among which line L114 showed the highest *vip3Aa* transcript accumulation (1,443.9-fold).

**Figure 6:**
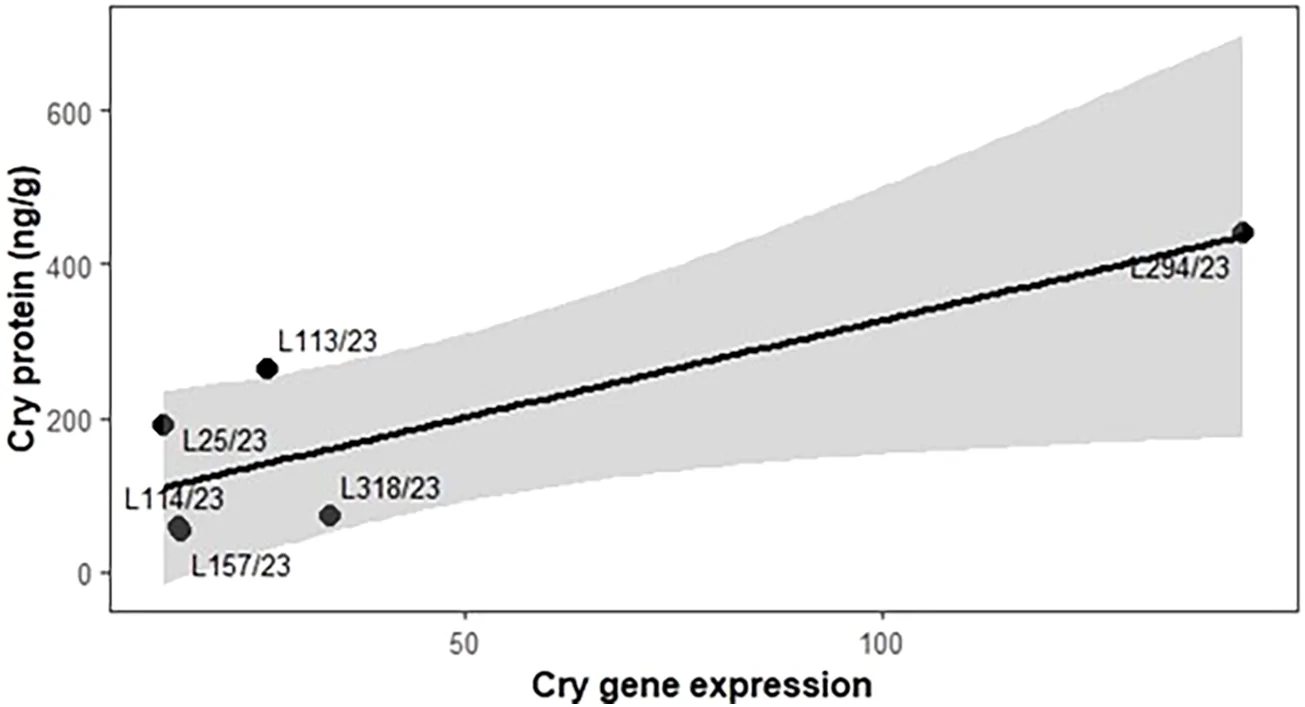
Correlation between *cry* gene expression and Cry protein accumulation. Scatter plot showing the relationship between relative *cry* transcript levels and Cry protein content (ng g^−1^ fresh weight) across selected transgenic sugarcane lines. The solid line represents the linear regression fit, and the shaded area indicates the 95% confidence interval. A significant positive correlation was observed (Pearson’s r = 0.83, p = 0.04).

To assess the relationship between transcript levels and protein accumulation, correlation analysis was performed between relative *cry1Ab* expression and Cry1Ab protein content (**Figure 6**). A strong positive correlation was observed (Pearson’s r = 0.83, p = 0.04), indicating a significant linear association between gene expression and protein accumulation across the evaluated lines.

## 4 Discussion

Sugarcane productivity is strongly constrained by biotic stresses, among which *D. saccharalis* represents one of the most damaging pests in the Americas. Conventional breeding, even when supported by molecular tools, faces important limitations for incorporating durable insect resistance into elite germplasm. These limitations are largely associated with the genetic complexity and long breeding cycles of sugarcane (Brant et al., 2025). Although host-plant resistance to *D. saccharalis* has been reported, it is generally quantitative and does not confer satisfactory protection, highlighting the need for complementary strategies such as Bt-based resistance (Tomaz et al., 2018).

In this context, genetic engineering provides a complementary approach to introduce insect resistance traits into elite germplasm. In the present study, transgenic events of the sugarcane variety TUC 03-12, were generated and evaluated. Events carrying either single *cry1Ab* genes or pyramided *cry1Ab* and *vip3Aa* constructs were successfully regenerated and evaluated under controlled conditions. Molecular analyses confirmed transgene expression, and phenotypic bioassays demonstrated, insecticidal activity against *D. saccharalis*.

Efficient genetic transformation of sugarcane depends on both a responsive tissue culture system and an effective gene delivery method. Particle bombardment remains widely used, transformation efficiencies are often low and highly genotype-dependent, particularly in elite cultivars (Singh et al., 2013; Weng et al., 2006; Wang et al., 2017). Recent advances have aimed to improve transformation efficiency and reduce genotype dependency (Wang et al., 2025). In the present study, transformation efficiency differed markedly between constructs, with *cry1Ab*-positive events recovered at a higher frequency than *vip3Aa*-containing events. This may reflect lower co-transformation or integration efficiency of the *vip3Aa* construct. This discrepancy likely reflects differences in construct size, gene complexity, and reduced regeneration efficiency associated with co-transformation events. Such limitations are commonly reported in biolistic transformation systems and may contribute to the lower recovery of pyramided lines.

Immunostrip-based lateral flow assays provide a rapid, cost-effective, and simple approach for screening Bt protein expression in transgenic plants and are therefore particularly useful as an initial step following PCR confirmation (Gao et al., 2016; Cristofoletti et al., 2018). In the present study, a high proportion of *cry1Ab*-positive lines showed detectable Cry1Ab protein, whereas Vip3Aa protein detection was restricted to a smaller subset of *vip3Aa*-positive events. This difference may be explained by post-transcriptional regulation, reduced protein stability, or lower translational efficiency, as previously reported for Vip proteins in tobacco (Kalia and Kaur, 2019) and cotton (Khan et al., 2020). Additionally, the inherent sensitivity limits of lateral flow assays may have contributed to the lower detection of Vip proteins, particularly in cases of low expression levels (Posthuma-Trumpie et al., 2009; Sajid et al., 2015).

Phenotypic bioassays conducted in controlled conditions demonstrated that most transgenic sugarcane lines induced significantly higher larval mortality than non-transgenic controls. The wild-type TUC 03–12 and non-transgenic regenerated control (line 22) exhibited low mortality rates, whereas the Bt maize control produced near-complete mortality, confirming the reliability of the bioassay system. Several transgenic lines achieved mortality levels above 80%, including both single-*cry1Ab* and pyramided events. Notably, some single-*cry1Ab* events performed similarly to that of pyramided events, suggesting that high levels of resistance can be attained without gene stacking under controlled conditions. Although synergism between insecticidal proteins is desirable, it is not required to delay the evolution of resistance, which was the main objective of pyramiding both proteins in sugarcane. Moreover, previous studies have shown that interactions between Cry and Vip proteins are context-dependent (Baranek et al., 2017; Nunes Lemes et al., 2014). In *D. saccharalis*, synergistic effects between Cry1 and Vip3 proteins were detected at LC_90_ but not at LC_50_, indicating that the magnitude of the interaction depends on both toxin concentration and the target (Nunes Lemes et al., 2014). In the case of transgenic events, protein expression is influenced by several factors, including the number and genomic position of insertions, as well as pre- and post-transcriptional regulatory mechanisms. Consequently, each generated event represents a unique combination of protein expression levels, and potential interactions, whether synergistic or antagonistic, should therefore be evaluated on an event-specific basis. In Brazil, the use of single-gene Cry sugarcane events has been reported as an effective component for lepidopteran pest management strategies, supported by high levels of field and experimental efficacy (de Oliveira et al., 2022). Nevertheless, pyramiding strategies remain important for resistance management since Cry and Vip proteins act through distinct receptors and modes of action, thereby reducing the likelihood of cross-resistance and contributing to trait durability (Carrière et al., 2016; Chakroun et al., 2016). Further studies comparing single-gene and pyramided events under equivalent expression levels will be required to determine the nature of their interaction in sugarcane against *D. saccharalis*. Quantitative analysis of Cry1Ab protein accumulation by ELISA revealed substantial variation among independent transgenic events, suggesting differences in transgene expression levels and/or protein stability. However, this variation was not clearly reflected in phenotypic bioassays, which generally showed high levels of larval mortality across most lines. The relatively narrow phenotypic response range under controlled conditions may have limited the detection of strong genotype–phenotype associations, despite the observed differences in protein accumulation (Vain et al., 2002; Rajeevkumar et al., 2015; Budeguer et al., 2021).

Gene expression analysis further supported this interpretation. Both *cry1Ab* and *vip3Aa* genes driven by the Zm-Ubi1 promoter exhibited overall high transcript accumulation, with relatively limited variation among transgenic lines. These findings are consistent with previous studies demonstrating strong and stable expression mediated by the Zm-Ubi1 promoter in monocot species (Wei et al., 2003; Guidelli et al., 2018). A significant positive correlation was observed between *cry1Ab* transcript abundance and Cry1Ab protein accumulation (r = 0.83, p = 0.04), indicating that transcriptional activity contributed substantially to protein levels in these events. This relationship agrees with previous reports linking Bt protein accumulation with enhanced insect resistance (Weng et al., 2011; Gao et al., 2016; Cristofoletti et al., 2018).

In contrast, *vip3Aa* expression and protein detection were more restricted and variable among pyramided events. Although several lines were confirmed to carry the *vip3Aa* transgene, only a subset exhibited detectable Vip3Aa protein accumulation, while RT-qPCR analyses revealed considerable differences in transcript levels across events. Notably, only three lines (L114, L157, and L294) consistently combined elevated transcript accumulation with detectable protein levels, suggesting more favorable expression profiles and highlighting promising candidates for further evaluation. Similar variability in Vip expression has been reported in other transgenic plant systems and has been associated with post-transcriptional regulation and/or reduced protein stability (Kalia and Kaur, 2019; Khan et al., 2020). Interestingly, previous studies in transgenic sugarcane expressing *vip3A* reported high levels of insect mortality against *Chilo infuscatellus* despite relatively moderate transcript accumulation (Riaz et al., 2020), suggesting that effective insect control may still be achieved at intermediate expression levels.

Overall, these results demonstrate the feasibility of generating transgenic sugarcane events harboring *cry1Ab* and *vip3Aa* genes, together with functional Bt gene expression and insecticidal activity against *D. saccharalis*. Cry1Ab-based constructs showed consistent performance across most evaluated lines, whereas Vip3Aa expression was more variable and restricted, emphasizing the need for further optimization of pyramided constructs and expression strategies. At present, most commercially available Bt sugarcane cultivars rely primarily on single Cry proteins, which may increase the risk of resistance evolution under prolonged field exposure. In this context, the development of pyramided Cry1Ab–Vip3Aa sugarcane represents a potentially promising approach to enhance the durability of insect resistance and support more sustainable pest management, although this will require validation under field conditions. These additional field evaluations will be required to validate agronomic performance, expression stability, and long-term efficacy, and the potential contribution of the pyramided traits to insect resistance management under commercial production conditions. The most promising events are currently undergoing vegetative multiplication to generate sufficient planting material for future field trials. These evaluations should also address resistance management and environmental biosafety, including potential effects on non-target organisms, gene flow, and the ecological interactions within sugarcane agroecosystems. Comprehensive risk assessment and regulatory evaluations will be essential before potential commercial deployment of Bt sugarcane events (de Oliveira et al., 2022; Lewis et al., 2025).

## Data Availability

The original contributions presented in the study are included in the article/**Supplementary Material**. Further inquiries can be directed to the corresponding author.

## References

[B1] Abbott W. S. (1925). A method of computing the effectiveness of an insecticide. J. Econ. Entomol. 18, 265–267. doi: 10.1093/jee/18.2.265a

[B2] Aljanabi S. M. Forget L. Dookun A. (1999). An improved and rapid protocol for the isolation of polysaccharide- and polyphenol-free sugarcane DNA. Plant Mol. Biol. Rep. 17, 281. doi: 10.1023/a:1007692929505 41886696

[B3] Altpeter F. Sandhu S. (2010). “ Genetic transformation – biolistics,” in Plant Cell Culture: Essential Methods. Eds. Davey M. R. Anthony P. ( Wiley, New York, NY), 217–237.

[B4] Baranek J. Konecka E. Kaznowski A. (2017). Interaction between toxin crystals and vegetative insecticidal proteins of Bacillus thuringiensis in lepidopteran larvae. Biocontrol 62, 649–658. doi: 10.1007/s10526-017-9828-6 30311153

[B5] Bernardi D. Bernardi O. Horikoshi R. J. Okuma D. M. Miraldo L. L. Fatoretto J. . (2015). Cross-resistance between Cry1 proteins in fall armyworm (Spodoptera frugiperda) may affect the durability of current pyramided Bt maize hybrids in Brazil. PLoS One 10, e0140130. doi: 10.1371/journal.pone.0140130 26473961 PMC4608726

[B6] Birch R. G. Bower R. S. Elliott A. R. (2010). Highly efficient, 5′-sequence-specific transgene silencing in a complex polyploid. Trop. Plant Biol. 3, 88–97. doi: 10.1007/s12042-010-9047-0 30311153

[B7] Bower R. Birch R. G. (1992). Transgenic sugarcane plants via microprojectile bombardment. Plant J. 2, 409–416. doi: 10.1111/j.1365-313x.1992.00409.x 40046247

[B8] Brant E. Zuniga-Soto E. Altpeter F. (2025). RNAi and genome editing of sugarcane: Progress and prospects. Plant J. 121, e70048. doi: 10.1111/tpj.70048 40051334 PMC11886501

[B9] Budeguer F. Enrique R. Perera M. F. Racedo J. Castagnaro A. P. Noguera A. S. . (2021). Genetic transformation of sugarcane: current status and future prospects. Front. Plant Sci. 12, 768609. doi: 10.3389/fpls.2021.768609 34858464 PMC8632530

[B10] Budeguer F. Racedo J. Enrique R. Perera M. F. Ostengo S. Noguera A. S. (2024). Transgenic sugarcane for the sustainable management of the sugarcane borer Diatraea saccharalis. Sugar Ind. 149, 133–138. doi: 10.36961/si30898

[B11] Carrière Y. Fabrick J. A. Tabashnik B. E. (2016). Can pyramids and seed mixtures delay resistance to Bt crops? Trends Biotechnol. 34, 291–302. doi: 10.1016/j.tibtech.2015.12.011 26774592

[B12] Chakroun M. Bel Y. Escriche B. (2016). Bacterial vegetative insecticidal proteins (Vip) from entomopathogenic bacteria. Microbiol. Mol. Biol. Rev. 80, 329–350. doi: 10.1128/mmbr.00060-15 26935135 PMC4867366

[B13] Cristofoletti P. T. Kemper E. L. Capella A. N. Carmago S. R. Cazoto J. L. Ferrari F. . (2018). Development of transgenic sugarcane resistant to sugarcane borer. Trop. Plant Biol. 11, 17–30. doi: 10.1007/s12042-018-9198-y 30311153

[B14] CTC (2025). Produtos Ctc. Available online at: https://ctc.com.br/produtos/ (Accessed February 11, 2025).

[B15] Cuenya M. I. Chavanne E. R. Ostengo S. Costilla D. D. García M. B. Díaz Romero C. . (2015). Comportamiento Productivo Y Fitosanitario De TUC 03-12, Una Nueva Variedad De Caña De Azúcar Para La Provincia De Tucumán (Tucumán, Argentina: Estación Experimental Agroindustrial Obispo Colombres). Gacetilla Agroindustrial No. 79.

[B16] D'Hont A. Grivet L. Feldmann P. Rao S. Berding N. Glaszmann J. C. (1996). Characterisation of the double genome structure of modern sugarcane cultivars (Saccharum spp.) by molecular cytogenetics. Mol. Gen. Genet. 250, 405–413. doi: 10.1007/BF02174028 8602157

[B17] de Oliveira W. S. Sakuno C. I. R. Miraldo L. L. Tavares M. A. G. Komada K. M. Teresani D. . (2022). Varied frequencies of resistance alleles to Cry1Ab and Cry1Ac among Brazilian populations of the sugarcane borer, Diatraea saccharalis (F.). Pest Manage. Sci. 78, 5150–5163. doi: 10.1002/ps.7138 36070208

[B18] Dively G. P. Venugopal P. D. Finkenbinder C. (2016). Field-evolved resistance in corn earworm to Cry proteins expressed by transgenic sweet corn. Proc. Natl. Acad. Sci. U.S.A. 113, 1973–1978. doi: 10.1073/pnas.1520362113 28036388 PMC5201267

[B19] Dyar H.G. Heinrich C. (1927). The American moths of the genus *Diatraea* and allies. Proc. U. S. Natl. Museum. 71, 1–48.

[B20] Fogliata S. V. Vera A. Gastaminza G. A. Cuenya M. I. Zucchi M. I. Willink E. . (2016). Reproductive isolation between two populations of Diatraea saccharalis (F.) (Lepidoptera: Crambidae) from different host plant species and regions in Argentina. Bull. Entomol. Res. 106, 591–597. doi: 10.1017/s0007485316000249 27112423

[B21] Gao S. Yang Y. Wang C. Guo J. Zhou D. Wu Q. . (2016). Transgenic sugarcane with a cry1Ac gene exhibited better phenotypic traits and enhanced resistance against sugarcane borer. PLoS One 11, e0153929. doi: 10.1371/journal.pone.0153929 27093437 PMC4836700

[B22] Garsmeur O. Droc G. Antonise R. Grimwood J. Potier B. Aitken K. . (2018). A mosaic monoploid reference sequence for the highly complex genome of sugarcane. Nat. Commun. 9, 2638. doi: 10.1038/s41467-018-05051-5 29980662 PMC6035169

[B23] Ghimire M. N. Huang F. Leonard B. R. Head G. P. Yang F. Niu Y. . (2018). Field-evolved resistance to Bt maize in sugarcane borer (Diatraea saccharalis) in Argentina. Pest Manage. Sci. 74, 905–913. doi: 10.1002/ps.4792 29095565 PMC5873414

[B24] Guidelli G. V. Mattiello L. Gallinari R. H. Lucca P. C. D. Menossi M. (2018). pGVG: a new Gateway-compatible vector for transformation of sugarcane and other monocot crops. Genet. Mol. Biol. 41, 450–454. doi: 10.1590/1678-4685-GMB-2017-0159 30088611 PMC6082244

[B25] Henríquez D. D. Figueroa M. F. Medina L. P. Díaz J. V. Díaz Romero C. Costilla D. D. . (2025). El rol crucial del Programa de Mejoramiento Genético de la EEAOC: diversificación varietal en el área cañera de Tucumán, Argentina en los últimos 10 años. Rev. Ind. Agric. Tucumán 102, 47–50.

[B26] Ingelbrecht I. L. Irvine J. E. Mirkov T. E. (1999). Posttranscriptional gene silencing in transgenic sugarcane. Dissection of homology-dependent virus resistance in a monocot that has a complex polyploid genome. Plant Physiol. 119, 1187–1198. doi: 10.1104/pp.119.4.1187 10198077 PMC32003

[B27] Kalia V. Kaur S. (2019). Efficacy of transgenic tobacco carrying a synthetic plant-preferred codon-optimized novel vip3Aa44 gene towards Helicoverpa armigera and Spodoptera litura. Indian J. Entomol. 81, 325–331. doi: 10.5958/0974-8172.2019.00054.3

[B28] Khan M. H. Mukhtar Z. Arshad M. Sarwar M. Asad S. (2020). Development and evaluation of synthetic vip3A gene in transgenic cotton for protection against chewing insect pests. Int. J. Agric. Biol. 25, 211–221. doi: 10.17957/ijab/15.1658 35601790

[B29] Kohli A. Twyman R. M. Abranches R. Wegel E. Stoger E. Christou P. (2003). Transgene integration, organization and interaction in plants. Plant Mol. Biol. 52, 247–268. doi: 10.1023/B.0000009265.18192.4c 12856933

[B30] Lewis D. M. Godoy P. Simeone F. (2025). Experiences, learnings and perspectives in the regulation of agricultural biotechnology: the view from Argentina. Front. Bioeng. Biotechnol. 13, 1600642. doi: 10.3389/fbioe.2025.1600642 40547298 PMC12179121

[B31] Livak K. J. Schmittgen T. D. (2001). Analysis of relative gene expression data using real-time quantitative PCR and the 2^−^ΔΔCt method. Methods 25, 402–408. doi: 10.1006/meth.2001.1262 11846609

[B32] Navure Team (2023). Navure (Version 1.2.0). Available online at: http://www.navure.com (Accessed February 11, 2025).

[B33] NK Semillas (2025). Ficha Técnica SYN 126 VIPTERA3. Available online at: https://nksemillas.com.ar/syn-126-viptera3 (Accessed February 11, 2025).

[B34] Noguera A. Enrique R. Perera M. F. Ostengo S. Racedo J. Costilla D. . (2015). Genetic characterization and field evaluation to recover parental phenotype in transgenic sugarcane: a step toward commercial release. Mol. Breed. 35, 115. doi: 10.1007/s11032-015-0300-y 30311153

[B35] Noguera A. Paz N. Díaz E. Perera F. Sepúlveda Tusek M. Filippone M. Castagnaro A. (2010). Proyecto Vitroplantas: La Producción De Caña Semilla De Alta Calidad Comienza En El Laboratorio (Las Talitas, Argentina: Estación Experimental Agroindustrial Obispo Colombres (EEAOC). Publicación Especial No. 40.

[B36] Nunes Lemes A. R. Davolos C. C. Legori P. C. B. C. Fernandes O. A. Ferré J. Lemos M. V. F. . (2014). Synergism and antagonism between Bacillus thuringiensis Vip3A and Cry1 proteins in Heliothis virescens, Diatraea saccharalis and Spodoptera frugiperda. PLoS One 9 (10), e107196. doi: 10.1371/journal.pone.0107196 25275646 PMC4183464

[B37] Ostengo S. Serino G. Perera M. F. Racedo J. Mamaní González S. Y. Yáñez Cornejo F. . (2022). Sugarcane breeding, germplasm development and supporting genetic research in Argentina. Sugar Tech. 24, 166–180. doi: 10.1007/s12355-021-00999-z 30311153

[B38] Perez M. L. D. P. Budeguer F. Isa R. B. Nikpay A. Gastaminza G. (2024). “ Main sugarcane pest management strategies in Argentina,” in Biotechnological Transformation for Sugarcane Management ( Apple Academic Press, Boca Raton, FL), 265–279.

[B39] Posthuma-Trumpie G. A. Korf J. van Amerongen A. (2009). Lateral flow (immuno)assay: its strengths, weaknesses, opportunities and threats. Anal. Bioanal. Chem. 393, 569–582. doi: 10.1007/s00216-008-2287-2 18696055

[B40] Qamar Z. Nasir I. A. Abouhaidar M. G. Hefferon K. L. Rao A. Q. Latif A. . (2021). Novel approaches to circumvent the devastating effects of pests on sugarcane. Sci. Rep. 11, 12428. doi: 10.1038/s41598-021-91985-8 34127751 PMC8203629

[B41] Rajeevkumar S. Anunanthini P. Sathishkumar R. (2015). Epigenetic silencing in transgenic plants. Front. Plant Sci. 6, 693. doi: 10.3389/fpls.2015.00693 26442010 PMC4564723

[B42] Riaz S. Nasir I. A. Bhatti M. U. Adeyinka O. S. Toufiq N. Yousaf I. . (2020). Resistance to Chilo infuscatellus (Lepidoptera: Pyraloidea) in transgenic lines of sugarcane expressing Bacillus thuringiensis-derived Vip3A protein. Mol. Biol. Rep. 47, 2649–2658. doi: 10.14264/uql.2018.172 32128710

[B43] Sajid M. Kawde A.-N. Daud M. (2015). Designs, formats and applications of lateral flow assay: A literature review. J. Saudi Chem. Soc 19, 689–705. doi: 10.1016/j.jscs.2014.09.001 38826717

[B44] Singh R. K. Kumar P. Tiwari N. N. Rastogi J. Singh S. P. (2013). Current status of sugarcane transgenics: an overview. Adv. Genet. Eng. 2, 112. doi: 10.4172/2169-0111.1000112

[B45] Tabashnik B. E. Brévault T. Carrière Y. (2013). Insect resistance to Bt crops: lessons from the first billion acres. Nat. Biotechnol. 31, 510–521. doi: 10.1038/nbt.2597 23752438

[B46] Tomaz A. C. Coutinho A. E. Soares B. O. Peternelli L. A. Pereira E. J. G. Barbosa M. H. P. (2018). Assessing resistance of sugarcane varieties to sugarcane borer Diatraea saccharalis Fab. (Lepidoptera: Crambidae). Bull. Entomol. Res. 108, 547–555. doi: 10.1017/s0007485317001183 29198198

[B47] Trumper E. V. (2014). Resistencia de insectos a cultivos transgénicos con propiedades insecticidas: teoría, estado del arte y desafíos para la República Argentina. Agriscientia 31, 109–126.

[B48] Vain P. James V. Worland B. Snape J. (2002). Transgene behaviour across two generations in a large random population of transgenic rice plants produced by particle bombardment. Theor. Appl. Genet. 105, 878–889. doi: 10.1007/s00122-002-1039-5 12582913

[B49] Wang W. Wang D. Zhao W. Zhang Y. Zeng Z. Hu Y. . (2025). Highly efficient and genotype-independent genetic transformation system in sugarcane. Plant Biotechnol. J. pp. 2241–2243. doi: 10.1111/pbi.70486 41348344 PMC13140336

[B50] Wang W. Z. Yang B. P. Feng X. Y. Cao Z. Y. Feng C. L. Wang J. G. . (2017). Development and characterization of transgenic sugarcane with insect resistance and herbicide tolerance. Front. Plant Sci. 8, 1535. doi: 10.3389/fpls.2017.01535 29033953 PMC5627015

[B51] Wei H. Wang M. L. Moore P. H. Albert H. H. (2003). Comparative expression analysis of two sugarcane polyubiquitin promoters and flanking sequences in transgenic plants. J. Plant Physiol. 160, 1241–1251. doi: 10.1078/0176-1617-01086 14610893

[B52] Weng L. X. Deng H. Xu J. L. Li Q. Wang L. H. Jiang Z. . (2006). Regeneration of sugarcane elite breeding lines and engineering of strong stem borer resistance. Pest Manage. Sci. 62, 178–187. doi: 10.1002/ps.1144 16408322

[B53] Weng L. X. Deng H. H. Xu J. L. Li Q. Zhang Y. Q. Jiang Z. D. . (2011). Transgenic sugarcane plants expressing high levels of modified cry1Ac provide effective control against stem borers in field trials. Transgenic Res. 20, 759–772. doi: 10.1007/s11248-010-9456-8 21046242

