# Supplementary material for: Transgenic sugarcane harboring cry1Ab and vip3Aa Bt genes for the management of Diatraea saccharalis
Source: Front Plant Sci. 2026 Aug 11;17:1887829. doi: 10.3389/fpls.2026.1887829 (PMC13503373; doi:10.3389/fpls.2026.1887829)
Supplement: Supplementary file 1 [file Table1.docx]

Supplementary Material

**Supplementary Figure 1.** **Phenotypic evaluation of transgenic sugarcane lines for resistance to D. saccaharlis under controlled conditions.** Abbot larval mortality (%) of the first 134 transgenic lines.
